# The multimorbidity epidemic: challenges for real-world research

**DOI:** 10.1017/S146342361900094X

**Published:** 2020-03-06

**Authors:** Muna Adan, Clare Gillies, Freya Tyrer, Kamlesh Khunti

**Affiliations:** 1 Pharmacoepidemiologist, Department of Health Sciences, University of Leicester, Leicester, UK; 2 Lecturer in Medical Statistics, Department of Health Sciences, University of Leicester, Leicester, UK; 3 Researcher in Epidemiology, Department of Health Sciences, University of Leicester, Leicester, UK; 4 Professor of Primary Care Diabetes and Vascular Medicine, University of Leicester, Leicester, UK; 5 Director, Leicester Diabetes Research Centre, Leicester, UK

Multimorbidity, defined as the co-occurrence of two or more long-term medical conditions in the same individual, continues to be a major health and social care challenge. Evidence shows that in England, multimorbidity accounts for 50% of general practice (GP) appointments, 64% of outpatient appointments, 70% of inpatient bed days and 70% of the total healthcare expenditure (Department of Health, 2012). From an individual perspective, multimorbidity reduces quality of life, physical functioning and life expectancy and increases the risk of psychological distress and the risk of adverse drug events from polypharmacy (Calderón-Larrañaga *et al*., 2012; Brettschneider *et al*., 2013; DuGoff *et al*., 2014).

The prevalence of multimorbidity is known to be increasing in the general population and is more common in the elderly population, in females and in individuals from low socioeconomic status (Barnett *et al*., 2012). Published prevalence studies have generally been few and have varied greatly in methodology. In observational studies, multiple morbidities have typically been determined using either health-based questionnaires/surveys or, more frequently, using electronic health records with the application of disease-specific medical codes. The main peril with the use of electronic health records is that there is no single standardized ‘correct’ code list that has been consistently used to ascertain any given morbidity and that a standard method of measuring multimorbidity does not exist (Barnett *et al*., 2012; Dhalwani *et al*., 2017). The codes, therefore, remain subjective and at the discretion of the researcher and clinical team involved in a study, meaning that they differ from one study to another and, inadvertently, affect the derived prevalence rates. In addition, multimorbidity is often defined in light of the number of conditions with little account of disease severity. Some morbidities are associated with worse disease outcomes than others, and certain clusters of morbidities occurring together may present more than an additive effect on future risk of complications or death (Dhalwani *et al*., 2016; 2017).

Patients with multiple long-term morbidities have traditionally been managed interchangeably between primary and secondary care, with diagnosis and treatment often instigated in secondary care, and patients’ follow-up conducted in primary care. However, over time and with the publication of a sequence of healthcare policies related to the management of long-term conditions, responsibility for chronic morbidities has increasingly shifted to primary care (Department of Health, 2006; 2008; Lester and Campbell, 2010). Conversely, this presents two immediate key obstacles. First, although as it currently stands, the GP contract in the UK sets consultation duration at 10 min, irrespective of the patient’s clinical profile, the national consultation average time has been estimated as being 8.86 min (Hobbs *et al*., 2016). Research conducted in England has highlighted that consultation times are compromised by the number of patients’ co-existing long-term conditions, showing a linear relationship of an average of 2 min for each extra problem (Salisbury *et al*., 2013). Therefore, it seems apparent that individuals with a greater number of long-term conditions need longer consultation times to cover the scope of care of each of the presenting complaints (inclusive of any therapeutic management), which could place an undue strain on general practitioners’ workload and resource use.

Secondly, the evidence base for delivering services for patients with multiple chronic long-term conditions in primary care is relatively sparse. A large systematic review identified only 10 studies that evaluated clinical interventions for improving outcomes in patients with multimorbidity. The review concluded that the evidence was limited, with many studies focusing on the effects of multimorbidity on patients and the healthcare system, as opposed to assessing interventions (Smith *et al*., 2012). Furthermore, despite the inception of several National Service Frameworks [of which the Quality Outcomes Framework is key], which have outlined the standards of primary care for specific numbers of long-term conditions, the management frameworks remain predominantly focused on ‘single-disease’ guidelines. This may limit the applicability of the recommendations to patients with multiple co-existing chronic conditions.

In addition, the use of multiple ‘single-disease’ guidelines could, unwittingly, increase unwarranted drug interactions, drug–disease interactions and drug–food interactions. For example, consider the clinical management of elderly patients with several co-existing chronic conditions such as osteoporosis, diabetes, hypertension, chronic obstructive pulmonary disorder and osteoarthritis. For patients to be compliant with all of the management recommendations from relevant guidelines, patients’ therapeutic and nontherapeutic treatment would consist of 12 pharmacological and 14 nonpharmacological regimens. Individually, this may seem rational and evidence-based, but the sum recommendation is not (Zulman *et al*., 2014). In light of this, the National Institute for Health and Care Excellence recently published recommendations on the assessment and management of patients with multimorbidity (The National Institute for Health and Care Excellence, 2016). However, given the dearth of research in this area of care, the recommendation remains markedly generic.

For example, the guideline has a general introduction and discussion on the importance of tailoring treatment and management to patients’ needs and preferences; however, there is little practical guidance on what this specifically entails or looks like. In addition, there is an emphasis on involving patients in the management decision-making process to ensure adherence of treatment; however, given the complex context of treatment-combinations, risks and benefit is not accounted for in the guidance nor is there a discussion on issues related to treatment failures.

It is apparent that a more integrated care response may be needed for people with multimorbidity. The concept, broadly defined and described in the literature, refers to an integration of interdisciplinary care across specializations that is incorporated into patients’ routine primary care with shared decision-making that results in a single combined long-term treatment plan adapted to address patients (complex) multitude of health problem (Trivedi, 2017; Rijken *et al*., 2018). The care builds in different working dimensions such as the type of integration, the extent of services involved and the level of integration needed.

It has further been argued that service users’ needs should be at the center of integrated care, under the rationale that patients with higher levels of needs as measured by degree of instability, chronicity of the condition, duality and complexity of treatments, and the general impact of the condition(s) would require a more comprehensive integrated health and, perhaps, social care approach than patients with lower levels of health needs (Rijken *et al*., 2018). Currently, at the service delivery level, the model that closely resembles the theoretical aspects of integrated care in practice is ‘case management’. This involves the identification of ‘at risk’ patients (typically defined in terms of at risk of hospital admission), assessment of their health needs and organizing and coordinating care between different healthcare professionals. However, although its delivery has been encouraged in the recent modifications to the National Health Service (NHS) (five-year framework) GP contract, its provision is purported to vary across the country and details of its practical application, particularly in patients with higher needs remain unclear (NHS England and British Medical Association, 2019).

NHS England’s ‘Long Term Plan’ report has outlined a pressing need to reshape the current delivery of care to improve national health outcomes (NHS England, 2019). We emphasize the importance of the healthcare system’s inclusion of a more holistic multidisciplinary model for patients with multimorbidity and further research to evaluate the outcomes.
